# Marginal zone lymphoma of palatine tonsil with prominent plasmacytic differentiation: A CARE-compliant article and review of literature

**DOI:** 10.1097/MD.0000000000009648

**Published:** 2018-01-12

**Authors:** Shuang Ma, Rachel Jug, Shuai Shen, Wan-Lin Zhang, Hong-Tao Xu, Lian-He Yang

**Affiliations:** a Department of Neurology, Sheng Jing Hospital of China Medical University, Shenyang, Liaoning; b Department of Pathology, Duke University Medical Center, Durham, NC; c Department of Pathology, First Affiliated Hospital and College of Basic Medical Sciences, China Medical University, Shenyang, Liaoning, China.

**Keywords:** differential diagnosis, mucosa-associated lymphoid tissue lymphoma, morphology, palatine tonsil, plasmacytic

## Abstract

**Rationale:**

The palatine tonsil is an important component of Waldeyer's ring and a site commonly involved by lymphoma. Interestingly, although it is a site of mucosa-associated lymphoid tissue (MALT), primary MALT lymphoma of the palatine tonsil is rare, especially with prominent plasmacytic differentiation.

**Patient concerns:**

A 59-year-old woman presented to the hospital with a 1-month history of odynophagia. The patient had no fever or pruritus during this period and she declared no family history of hematolymphoid malignancy.

**Diagnosis:**

Histopathological examination demonstrated effacement of tonsil architecture; normal follicles were replaced by plasmacytoid tumor cells and small lymphocytes. The tumor cells expanded the marginal zone and infiltrated interfollicular regions, as well as scattered residual follicles. Immunostaining showed tumor cells positive for cluster of differentiation (CD)20, CD79a, paired box-5, Mum 1, and B cell lymphoma (Bcl)-2, and negative for CD5, CD 23, cyclin D1, Bcl-6, and CD10. Staining for κ and λ showed prominent light chain restriction. The tumor was classified as tonsil MALT lymphoma with prominent plasmacytic differentiation.

**Interventions:**

After the patient was diagnosed with MALT lymphoma with prominent plasmacytic differentiation, she underwent complete surgical resection and radiotherapy.

**Outcomes:**

There was no recurrence evident at 6-months follow-up.

**Lessons:**

Primary tonsil MALT lymphoma with prominent plasmacytic differentiation is very rare and difficult to distinguish from other B-cell lymphomas with plasmacytoid morphology, such as follicular lymphoma, lymphoplasmacytic lymphoma, and chronic lymphocytic leukemia/small lymphocytic lymphoma. Accurate diagnosis of this entity is important in guiding therapy so as to avoid overtreatment.

## 1 Introduction

The palatine tonsils are located at the intersection of the digestive tract and the respiratory tract, where mucosa contains abundant lymphoid tissue and is exposed to foreign antigens. As with other lymphoglandular tissues comprising the body's immune system, tonsils serve a primary role in helping the body fight infection. Most lymphomas found in the palatine tonsils are of the B-cell type; and, of these, diffuse large B-cell lymphoma (DLBCL) represents most of the cases, reported to comprise up to 80% in some studies.^[1–5]^ Lymphoma of mucosa-associated lymphoid tissue (MALT) arising from the tonsil is a rare phenomenon.^[3,4,6–8]^ Morphologically, lymphoma cells may be small to medium sized, presenting as small lymphocytes or with a monocytoid appearance. One-third of gastric MALT lymphomas display plasmacytic differentiation which is also frequently seen in cutaneous and thyroid MALT lymphomas, but very rare in tonsil MALT lymphomas. To the authors’ knowledge, the current case is the 2nd report of primary MALT lymphoma with prominent plasma cell differentiation affecting the palatine tonsil.

## 2 Case presentation

A 59-year-old woman presented to the hospital for a 1-month history of odynophagia. She did not experience concurrent fever or pruritis, nor did she have a personal history of malignancy or other serious illness. The patient had not received any prior therapy for lymphoma. She had no family history of tumors affecting the lymphatic or hematopoietic systems. Fiberoptic laryngoscopic examination and computed tomographic imaging revealed a left tonsillar lesion. Pathological examination was performed and a diagnosis of primary tonsil MALT lymphoma with prominent plasmacytic differentiation was made.

## 3 Materials and methods

The resected tumor samples were embedded in paraffin blocks and serially sectioned. Immunohistochemistry was performed using an SP kit (Maixin Biotechnology, Fuzhou, Fujian, China) according to the manufacturer's instructions. The sections were incubated overnight at 4°C with the following primary antibodies: B cell lymphoma (Bcl)-2 (1:200, Dako, Carpinteria, CA), Bcl-6 (1:100, Dako), cluster of differentiation (CD)10 (1:100, Dako), CD138 (1:100, Dako), CD3 (1:100, Dako), CD20 (1:100, Dako), κ (1:100, Dako), λ (1:100, Dako), CD56 (1:200, Dako), Ki-67 (1:200, Dako), Mum-1 (1:100, Dako), and paired box (Pax)-5 (1:100, Dako). Immunostaining showing a granular brown substance in the appropriate subcellular locations was considered positive. This study was prospectively performed and approved by the institutional Ethics Committees of China Medical University and conducted in accordance with the ethical guidelines of the Declaration of Helsinki. Written informed consent was obtained from the patient for the publication of this case report and accompanying images.

## 4 Results

### 4.1 Gross features

Radiologic examination of the neck did not detect lymphadenopathy. Fiberoptic laryngoscopic examination demonstrated a bulging left tonsil. Tonsillectomy was performed and the specimen was submitted for pathological examination. The tonsil weighed 11 g and measured 3.6 × 3.0 × 2.1 cm. The mucosa was pink-red and smooth. The tonsil was serially sectioned to reveal a homogeneous pink-tan cut surface.

### 4.2 Microscopic features

**Figure 1:**
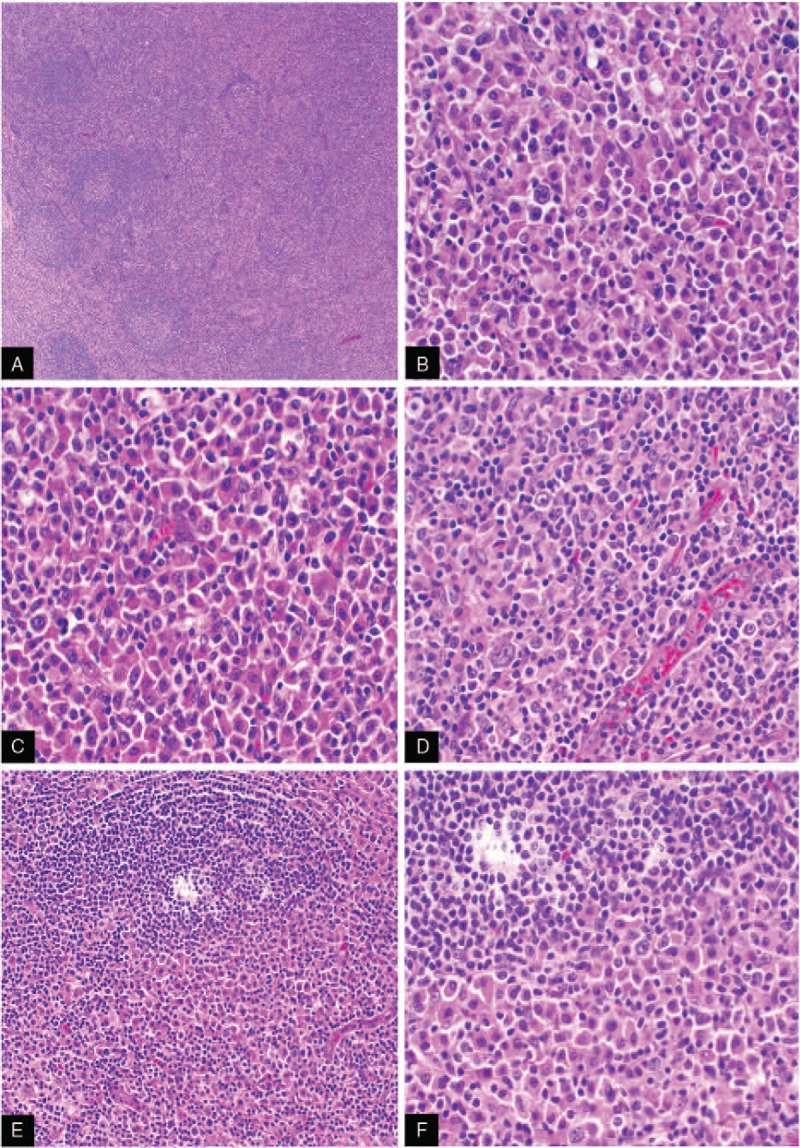
(A) Scattered residual normal follicles in the background of serious destruction of normal tonsil architecture (4×); (B) normal structure of the tonsil was replaced by numerous tumor cells which present prominent plasmacytic differential and atypical features (40×); (C and D) the plasmacytic tumor cells expand the marginal zone of the residual follicle and infiltrate into perifollicular region, and the great atypical plasmacytic cells mixed with small population of lymphocyte (40×). (E) (20×) and (F) (40×) normal structure of follicle was broken by the plasmacytic tumor cells, only scattered germinal center cells could be found under high magnification.

Histologically, the luminal surface of normal tonsils is covered with a stratified squamous epithelium. Beneath the epithelium, there are abundant lymphoid follicles with germinal centers similar to those seen in lymph nodes. Microscopic examination of our case revealed marked disruption of the normal tonsil architecture. From low magnification, effacement germinal centers were evident with only scattered residual germinal centers seen (Fig. 1A). Tonsillar tissue was replaced by eosinophilic plasmacytoid tumor cells. Examination of the tumor cells at high magnification showed numerous atypical plasmacytoid cells with a heterogeneous size and shape. The nuclear contour was irregular yet prominent and the nucleus contained a large nucleolus. The cytoplasm exhibited strong eosinophilic staining (Fig. 1B), scattered large atypical cells could be found under high magnification (Fig. 1C and D), the neoplastic B-cells assumed an interfollicular growth pattern, plasmacytoid tumor cells invaded and effaced follicles, residual mantle zone lymphocyte could be found. Marginal zone cells, parafollicular cells, and germinal center cells were replaced by plasmacytic tumor cells (Fig. 1E and F).

### 4.3 Immunophenotypic studies

**Figure 2:**
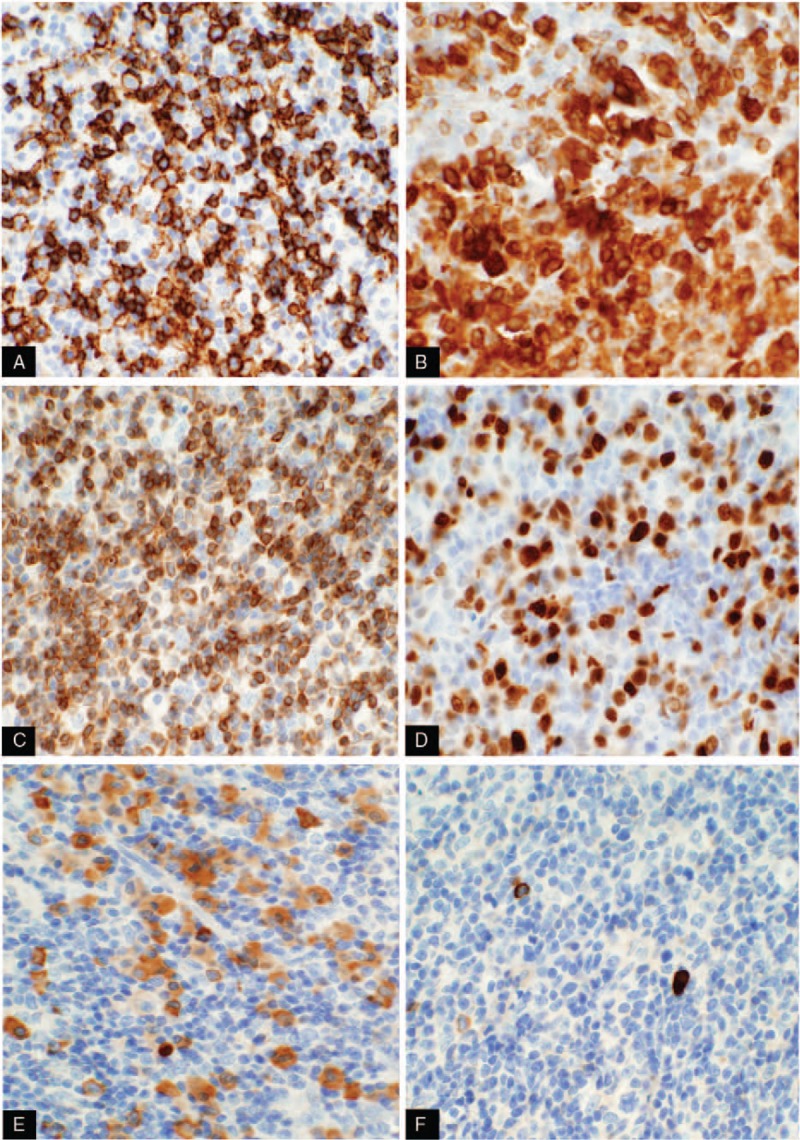
(A) CD20: highlights lymphoid follicles and increased interfollicular plasmacytic tumor cells (20×); (B) CD79a highlights lymphoid follicles and increased interfollicular B cells, including large plasmacytoid cells (20×); (C) Bcl-2 highlights staining in large plasmacytoid cells (40×); (D) Mum-1 is positive in plasmacytoid cells in perifollicular areas (40×). (E) κ stains plasmacytoid cells in interfollicular areas, including many tumor cells with abundant cytoplasm (40×); (F) λ stains scattered rare plasma cells that appear benign (40×). Bcl = B cell lymphoma, CD = cluster of differentiation.

CD20 highlights lymphoid follicles and increased interfollicular B cells, as well as scattered large cells (Fig. 2A). CD79a highlights lymphoid follicles and increased interfollicular B cells, including large plasmacytoid cells (Fig. 2B). Bcl-2 stains interfollicular cells and mantle zone cells, but is negative in germinal centers (Fig. 2C). Mum-1 is positive in plasmacytoid cells in interfollicular or perifollicular areas (Fig. 2D). Pax-5 highlights lymphoid follicles and increased interfollicular B cells with weak staining of large plasmacytoid cells. CD3 and CD5 highlight interfollicular T cells; CD10 and Bcl-6 stain for residual follicular centers cells. Ki67 is approximately 10% in the hot area. κ stains plasmacytoid cells in interfollicular areas, including many tumor cells with abundant cytoplasm (Fig. 2E), whereas λ stains scattered rare plasma cells that appear benign (Fig. 2F), the ratio of κ/λ staining is >10:1. CD138 stains plasmacytoid cells, CD56 is partially positve in plasmacytoid cells.

## 5 Discussion

Lymphomas encompass a complex group of malignancies and account for the 2nd commonest group of neoplasms of the head and neck after squamous cell carcinoma.^[9]^ Non-Hodgkin's lymphoma can involve any region of the head and neck, however, Waldeyer's ring, nasal cavities, and paranasal sinuses together account for 90% for all extranodal lymphoma sites in the head and neck. Among the aforementioned areas, Waldeyer's ring (including tonsil, nasopharynx, and base of tongue) is the most commonly involved extranodal site. Within the Waldeyer's ring, more than 50% of lymphomas arise in the palatine tonsil.^[1,4,8–15]^

Most lymphomas involving the tonsil are non-Hodgkin's type, and approximately 85% are of diffuse large B-cell type.^[4,10,13,14,16–18]^ Most of non-Hodgkin's lymphomas in the tonsil are of B-cell lineage, including DLBCL, follicular lymphoma (FL), mantle cell lymphoma, lymphoplasmacytic lymphoma, plasmacytoma, and extranodal MALT lymphoma. Interestingly, the occurrence of MALT-type lymphomas in the tonsils is rare despite the substantial amount of MALT encountered at this location.^[1,8,13,15,18]^

MALT is characterized by reactive follicles with prominent marginal zones, infiltration of overlying epithelium by lymphocytes, and the presence of subepithelial plasma cells. In contrast, MALT lymphoma can present with proliferation of marginal zone B cells, composed of morphologically heterogeneous small B cells including centrocyte-like cells, monocytoid cells, small lymphocytes, and plasma cells. In this case, the tumor cells presented predominantly with plasmacytic differentiation. This morphology has been reported in approximately one-third of gastric MALT lymphomas and is frequently seen in cutaneous MALT lymphomas. Surprisingly, MALT lymphomas very rarely present with plasmacytic differentiation in the tonsil. To the best of our knowledge, this is the 2nd case of MALT lymphoma reported in the English literature which occurred in palatine tonsil and showed predominantly plasmacytic differentiation.

Plasmacytic differentiation is not a specific feature of MALT lymphoma. When plasmacytic differentiation is identified as a predominant feature, several lymphomas should be considered in formulating a differential diagnosis, including lymphoplasmacytic lymphoma (LPL), chronic lymphocytic leukemia/small lymphocytic lymphoma (CLL/SLL), FL, and extramedullary plasmacytoma (EMP).^[19–22]^ LPL is a rare neoplasm of older patients (50–69 years) with involvement of bone marrow, lymph node, spleen, and liver. To our best knowledge, LPL occurring in the tonsil as the primary site has not been reported. Most patients have monoclonal immunoglobin M and Waldenström macroglobulinemia with hyperviscosity symptoms, which were not apparent in our case. Morphologically, LPL is composed of small lymphocytes, plasmacytoid lymphocytes, and plasma cells, and usually involves the bone marrow, although not uncommonly is seen in lymph nodes and/or the spleen. Typical features such as Dutcher bodies, increased mast cells, and hemosiderin were not identified in our patient. The characteristic immunophenotype includes positive staining for B-cell antigens, and coexpression of Pax-5 and CD138-positive plasma cells.^[23,24]^ CLL/SLL is extremely uncommon in the tonsil, especially at the time of initial presentation of the disease.^[9]^ SLL often presents with leukemia (CLL), although patients may be asymptomatic. Morphologically, it is characterized by a proliferation of small round lymphocytes interspersed individually with prolymphocytes and paraimmunoblasts. Tumor cells typically involve nodal or extranodal tissues as a diffuse proliferation with pseudofollicular growth centers, which easily mimic the growth pattern of MALT lymphoma. Plasmacytic differentiation is rarely found in CLL/SLL cases, and expression of CD5 and CD23 is useful to distinguish CLL/SLL from MALT lymphoma. FL showing marked plasmacytoid differentiation appears to be extremely rare, making up approximately 3.5% of cases. In such cases, the growth pattern shows plasmacytic tumor cells infiltrating follicles and interfollicular areas. An extensive workup including immunostaining and molecular genetic studies to completely distinguish FL with plasmacytoid differentiation from MALT lymphoma. Identification of the small attenuated CD10^+^/Bcl-2^+^ neoplastic follicles and positive immunoglobulin H*/BCL2* fusion by fluorescence in situ hybridization are important features for diagnosing FL in addition to the vast majority also being positive for t (14; 18) resulting in overexpression of the Bcl-2 gene. Conversely, negative expression of Bcl-2 excludes the diagnosis of FL.^[25]^ EMP occurs outside of the bone marrow is uncommon, most of these cases involve in the head and neck region (80%), such as nasopharynx and sinonasal cavities, while tonsil is a very uncommon site which could be rarely get involved.^[21,22]^ Histologically, plasmacytomas are composed of mature, immature, plasmablastic or anaplastic plasma cells. In the current case, residual germinal centers and small lymphocytes do not support a diagnosis of EMP, other morphological feature such as: mott cells/morula cells, Russell body, flame cells, Gaucher-like cells/thesaurocytes, cytoplasmic crystals, and Dutcher bodies were absent in current case. Immunostaining showing positive expression for Bcl-2 is also very helpful to exclude the diagnosis of EMP.

Our case presented with tumor cells infiltrating of the marginal zone of reactive B-cell follicles and extension into the interfollicular region. The tumor cells exhibit prominant plasmacytic differentiation and a classic immunophenotype consistent with the diagnosis of MALT lymphoma with prominent plasma cell differentiation. According to the previous report, the prognosis of primary tonsil lymphoma is excellent. A treatment regimen comprised of combined chemotherapy and radiation therapy is well tolerated, highly effective, but may result in overtreatment of patients. MALT lymphomas are optimally treated using local modalities, such as radiation and/or surgical resection, with most patients having a good outcome,^[26]^ but it still needs to be further studied to avoid overtreatment of this generally low-grade lymphoma. In summary, attention should be paid to identify MALT lymphoma in the tonsil, especially cases with prominent plasmacytic differentiation.
